# Supplementary material for: Pancreatic Islet Viability Assessment Using Hyperspectral Imaging of Autofluorescence
Source: Cells. 2023 Sep 19;12(18):2302. doi: 10.3390/cells12182302 (PMC10527874; doi:10.3390/cells12182302)
Supplement: Supplementary file 1 [file cells-12-02302-s001.zip › cells-2591282-supplementary.pdf]

Supplementary materials

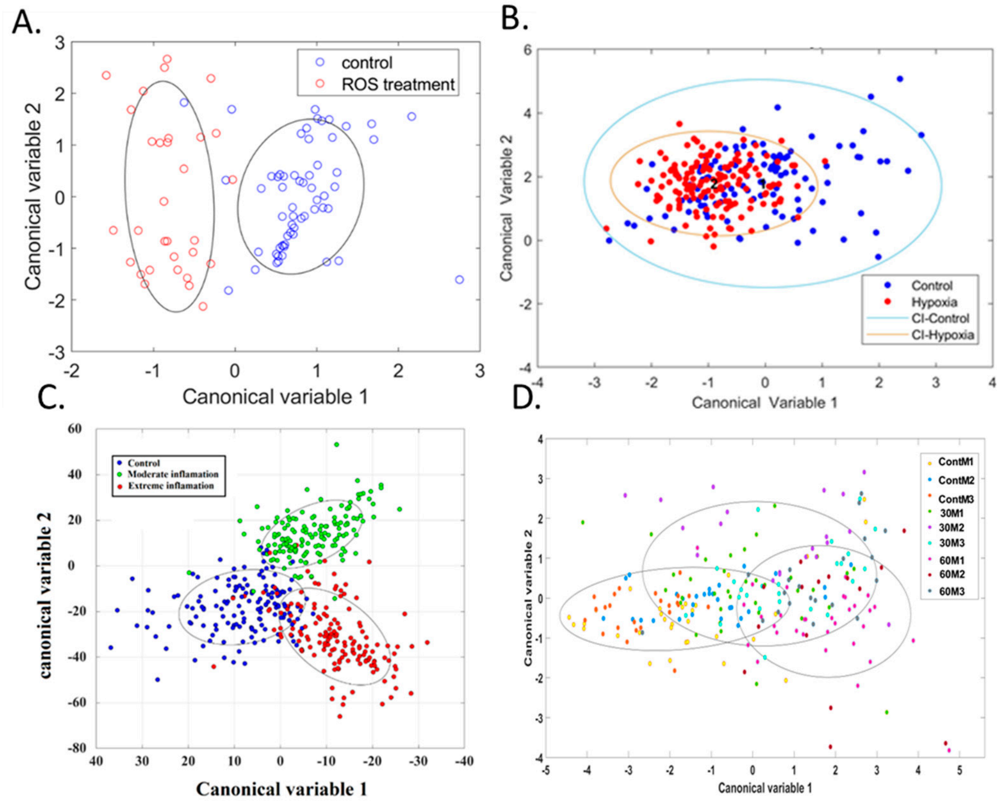

Supplementary figure 1: Cluster analysis of cells from islets affected by A) ROS damage, B) hypoxia, C) inflammatory cytokines, and D) warm ischemia. Data points represents individual cells, ellipses encompass 1 standard deviation around the mean. In D individual colours correspond to mouse of origin as indicated in the legend, with ContM1, 2 and 3 indicating cells from control islets, 30M1, 2 and 3, indicating cells from islets exposed to moderate warm ischemia (30 minutes), and 60M1, 2 and 3 indicating cells from islets exposed to extreme warm ischemia (60 minutes).

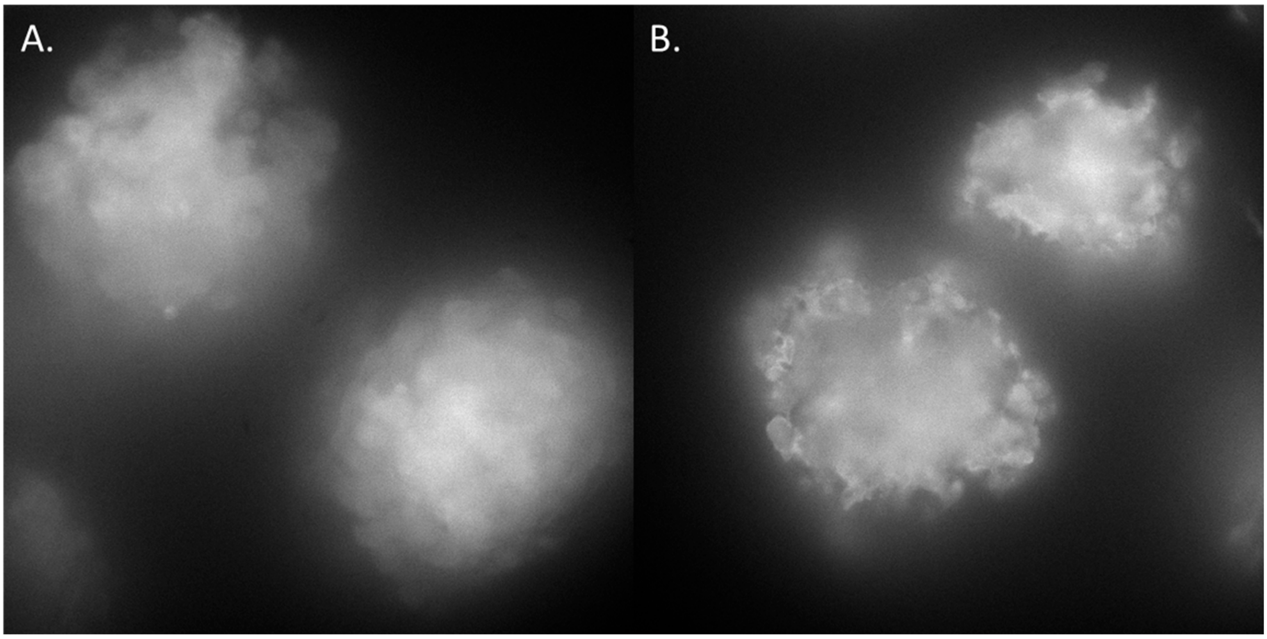

Supplementary figure 2. Representative images of islets subjected to the encapsulation process. A) Unencapsulated islets which were taken through the process without the inclusion of the encapsulation material, B) Encapsulated islets.

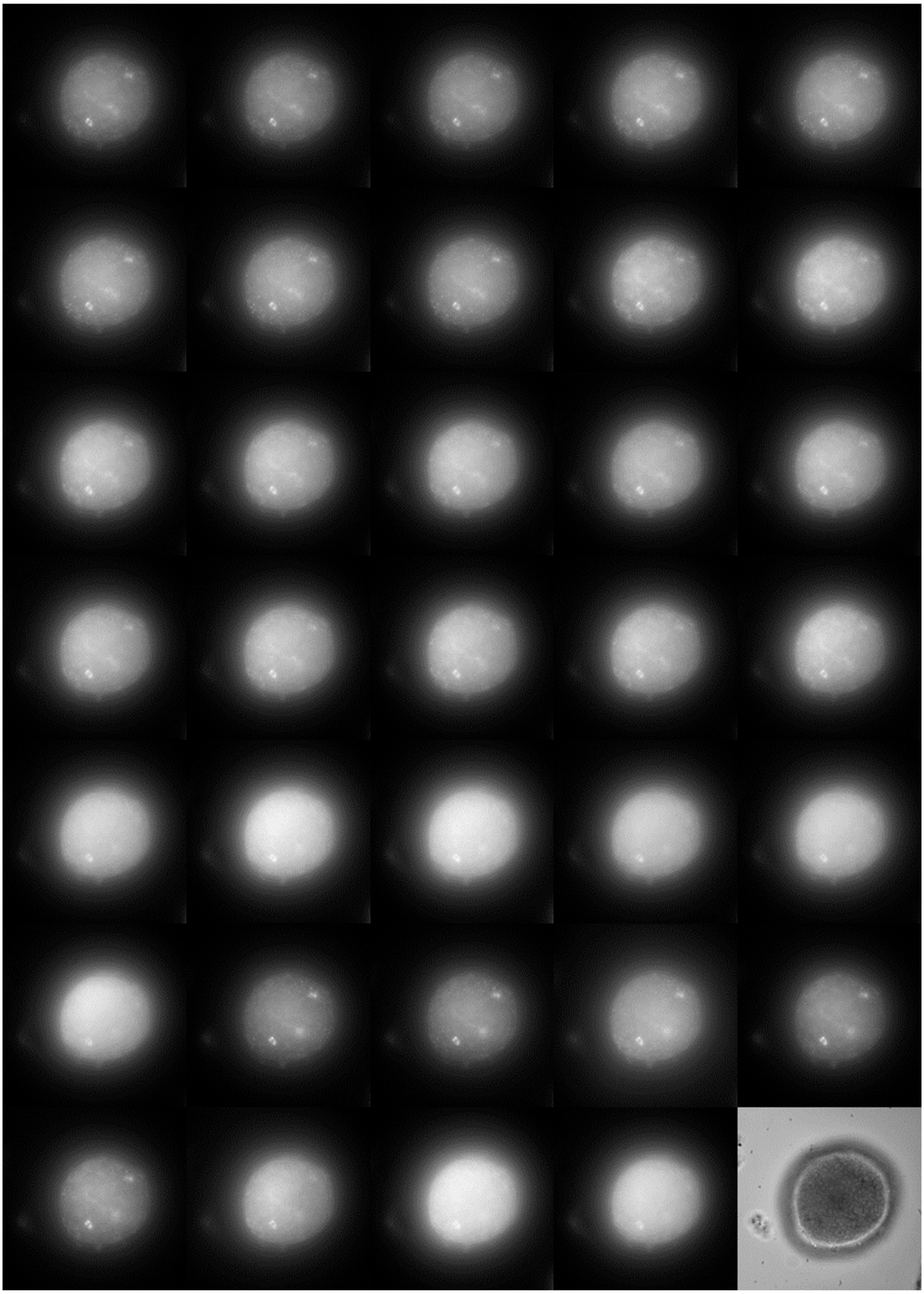

Supplementary figure 3. A full spectral stack of a pristine islet. Working from left to right, top to bottom, images represent the excitation/emission (nm) combinations: 325/414, 325/451, 325/ 575, 431/575, 438/575, 325/594, 431/594, 438/594, 339/414, 343/414, 356/414, 343/457, 356/451, 366/451, 339/575, 343/575, 356/575, 366/575, 373/575, 377/575, 381/575, 384/575, 388/575,

393/575, 403/575, 408/575, 388/594, 393/594, 396/594, 400/594, 403/594, 408/594, 414/594, 425/594, Brightfield.

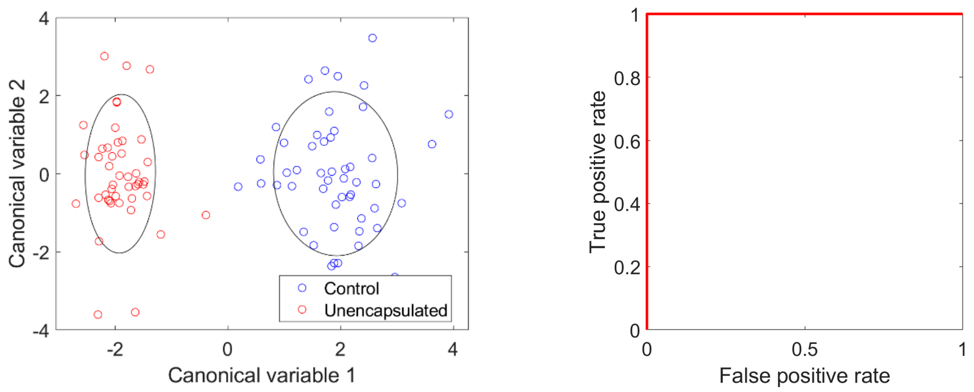

Supplementary figure 4: Islets taken through the encapsulation process without encapsulation material (red) compared to control islets not treated in any way (blue). AUC =1.0

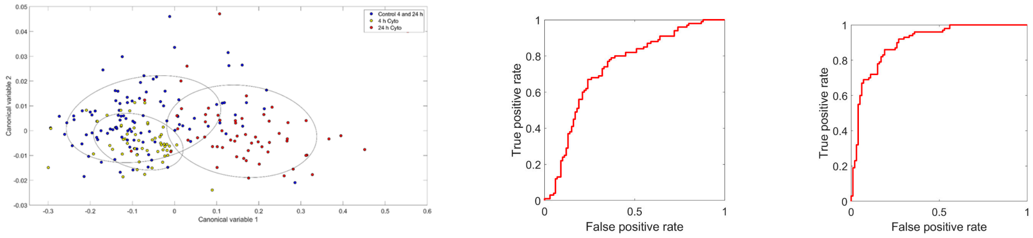

Supplementary figure 5: Encapsulated islets exposed to pro-inflammatory cytokines for 4 and 24 hours, discrimination of pristine and 4h AUC= 0.73, discrimination of pristine and 24 hour AUC = 0.89

Supplementary material 1

Unmixing single cells

Using unmixing assessment we were successfully able to identifying the spectral signals for NAD(P)H, Flavins, collagen and cytochrome C. Most signals were isolated for all conditions, however the signal

for cytochrome C could not be reliably identified for the cytokine model and has not been reported. Additionally the redox ratio (NAD(P)H/Flavins), a common measure of cell metabolic state was also calculated.

NAD(P)H

Level of NAD(P)H was not significantly impacted by ROS accumulation although it was significantly elevated by hypoxia. In the assessment of inflammatory cytokine exposure NAD(P)H was significantly elevated after 4 hours of exposure compared to cells from islets that had been maintained in normal culture for 4 hours, however the difference between cells from islets exposed for 24 hours and islets that had been cultured normally was not significant. This effect is likely due to the significant elevation in NAD(P)H that was observed to occur with time as shown by the significant difference between the 4 and 24 hour control groups. Finally, NAD(P)H was significantly elevated in cells from islets exposed to 60 minutes of warm ischemia compared to 30 minutes of warm ischemia, but neither group was significantly different compared to controls.

Flavins

Levels of flavins in cells were not affected by islets' exposure to ROS, hypoxia or pro-inflammatory cytokines, however they were significantly reduced after 60 minutes of warm ischemia relative to 30 minutes of exposure to warm ischemia.

Redox ratio

Cell redox ratio (RR) is a standard measure of metabolic state and is calculated here as NAD(P)H/Flavins. It was not significantly affected by ROS exposure, but was increased by hypoxia. Additionally, it was elevated in cells from islets exposed to inflammatory cytokines for 24 hours relative to 4 hours of exposure as well as cells from 24 hour control islets relative to 4 hour controls. For warm ischemia RR was elevated in cells from islets exposed to 30 or 60 minutes of warm ischemia.

Collagen

No treatments showed any significant differences in collagen levels. This was not surprising due to the extracellular matrix having been destroyed by EDTA in order to disaggregate islets to single cells.

Cytochrome C

Unmixing is most effective on the most dominant fluorophores which make up the overall autofluorescent output of a cell – the most consistent of these being NAD(P)H and flavins. Consequently there can be variation between experiments in which fluorophores can be distinguished. For the cytokine investigation the cytochrome C signal was not clear and therefore has not been reported. Levels with ROS and hypoxia did not differ significantly, however warm ischemia resulted in significant reductions at 60 minutes compared to controls and 30 minutes.

Supplementary material 2

Unmixing methodologies

The identification of fluorophoresis done by comparing the endmember spectra found in the islets to the reference spectra of pure fluorophores (measured in a separate in vitro experiment, as explained in [32, 20, 39, 53]. We have compared four endmembers which were the most similar to their respective reference fluorophores (NAD(P)H, flavin, Collagen I, cytochrome c). As per previously published literature [1-4] NADH and flavins are universally observed and they were observed in the islets. Other fluorophores we found (cyt C and collagen 1) have also been successfully identified by autofluorescence unmixed in our published work [32, 20]. The accuracy of RODECA in determining fluorophore spectra and abundancies is discussed in [20]. It is determined by the signal to noise ratio which is comparable in these data and in our other published works [32, 20, 39, 53] as we used broadly similar experimental conditions.

In this analysis RoDECA was used for unmixing the hyperspectral data in identifying four fluorophores: NAD(P)H, FAD, cytochrome c and collagen I. Here, we show a proof of concept of unmixing, by using a semi-supervised unmixing algorithm to show that we can extract fluorophores. Minimum volume simplex analysis (MVSA) was used to extract fluorophores. This unmixing algorithm is comparable with RoDECA [4]. Tan et al. [53] shows how RoDECA and MVSA can be used to extract fluorophores, where here we used similar pre-processing steps to perform dimensionality reduction, decorrelating data and optimising the vector subspace to project the data. Supp Fig 6 compares the reference (in red) and extracted spectra (in black) for (a) NAD(P)H, (b) FAD, (c) cytochrome c and (d) collagen I. The same principle was used when examining extracted spectra from RoDECA, to confirm that we were able to identify NAD(P)H, FAD, cytochrome c and collagen I.

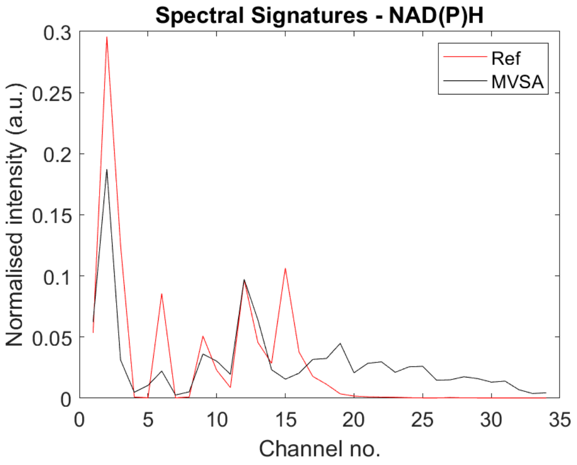

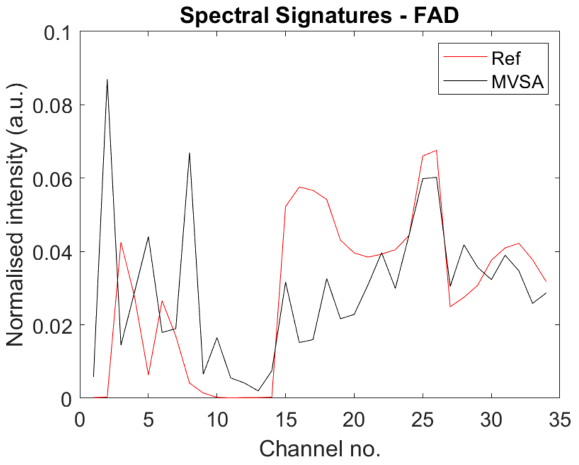

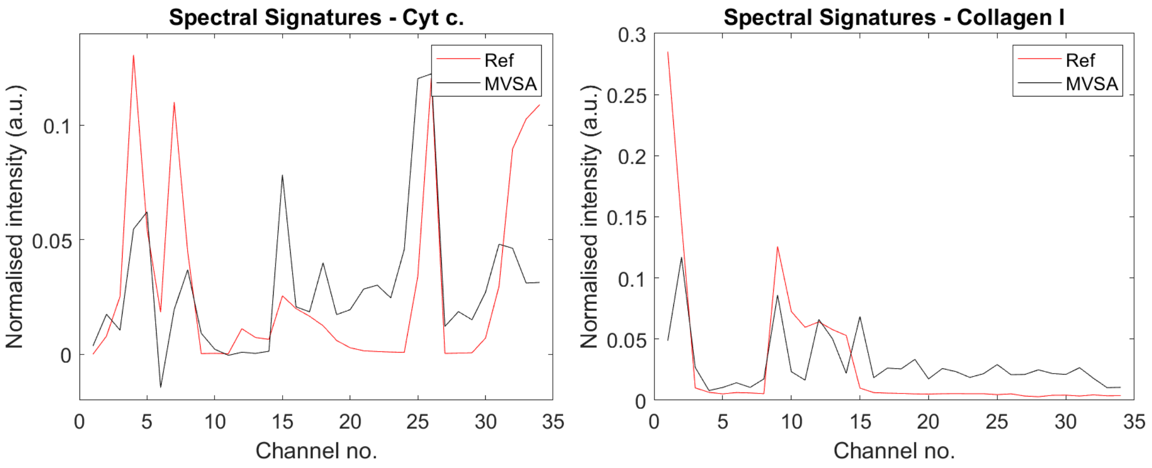

Supp fig 6. Comparison of reference (red) and extracted (black) spectra of fluorophores NAD(P)H, FAD, Cytochrome c and Collagen I from islet data.
